# Coopetitive Supply Chain Relationship Model: Application to the Smartphone Manufacturing Network

**DOI:** 10.1371/journal.pone.0132844

**Published:** 2015-07-17

**Authors:** Jeremy Jie Ming Kwok, Dong-Yup Lee

**Affiliations:** 1 Department of Chemical and Biomolecular Engineering, National University of Singapore, Singapore, Singapore; 2 Graduate School for Integrative Sciences and Engineering, National University of Singapore, Singapore, Singapore; 3 Bioprocessing Technology Institute, Agency for Science, Technology and Research (A*STAR), Singapore, Singapore; Korea University, REPUBLIC OF KOREA

## Abstract

Previous researches for understanding supply chain relationship have mostly focused on its vertical collaboration between buyers and suppliers. However, there have been some instances of volatile and stable collaborative relationships amongst competitors such as Apple-Samsung product manufacturer-component supplier relationship and airline alliances, respectively, which is recognized as coopetition. Even though there have been several qualitative studies and a number of game theory models on coopetition, it is rare to find any attempts on quantitative characterization of such coopetitive dynamic behavior in supply chain relationship. Hence, in this work, we formulated a MINLP model mathematically representing coopetitive relationships in a cost efficient supply chain network. In particular, the coopetition factor was newly introduced to measure the degree of coopetition among supply chain players and determine the optimal level of coopetition to engage in. The utility and practicality of the model were strongly demonstrated using a case study of a hypothetical smartphone supply chain network under different scenarios, thus proposing their strategically viable optimal interactions. Therefore, this exploratory study can herald a new era of global coopetitive business.

## Introduction

“Supply Chain 2.0” has to be lean, nimble and structurally flexible [1] in this fast-paced global economy, thus emphasizing the importance of strong supply chain relationship. Such studies have focused on vertical cooperation between buyers and suppliers, exploring its impact on firm performance [2, 3] and the crucial factors [4, 5] and impediments [6] to its success. However, in some cases, there are also collaborations among competitors as exemplified by the cooperative relationship between Apple and Samsung before their recent legal dispute [7], where Samsung supplied majority of the components for Apple's iPhone [8, 9] while both companies compete in the smartphone consumer market. Another example, outside of the supply chain context, is airline alliances where the firms strategically form the stable global network to broaden their flight connectivity, thereby giving travelers more convenience [10]. This simultaneous interaction of cooperation and competition is called coopetition [11, 12] which may be fragile or strong depending on various business circumstances. Despite several qualitative studies on inter-firm coopetition, only a hand of works have discussed such phenomenon in supply chains [13].

**Fig 1:**
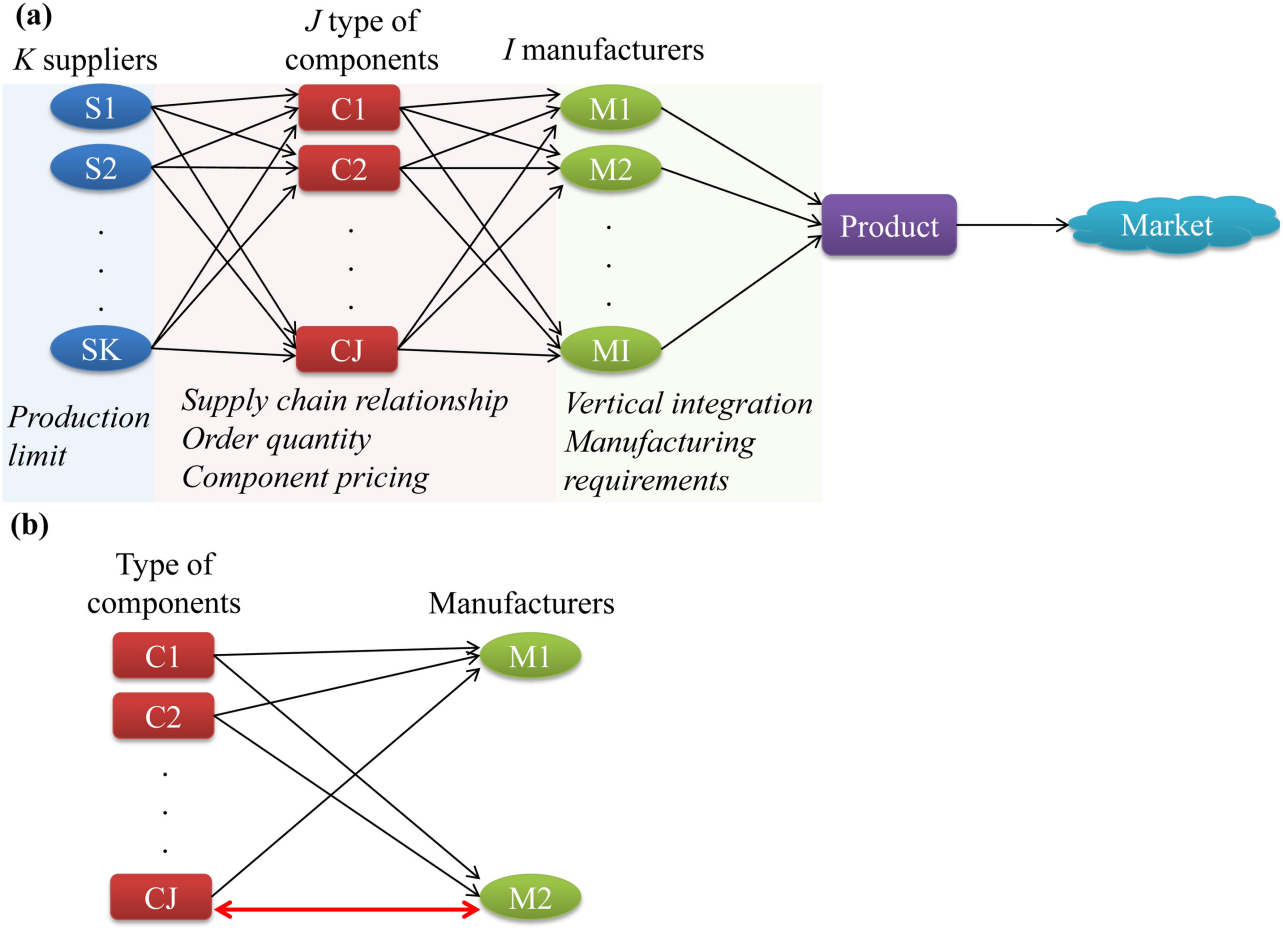
Illustration of coopetitive supply chain relationship. (a) General manufacturing supply chain network; (b) Coopetition due to vertically integrated company M2 which supplies M1 with component CJ; Red bidirectional arrow points to the components which M2 produces and sells to other manufacturers.

In a manufacturing supply chain, where component suppliers enter contracts with end-product manufacturers (OEMs) to provide them with various types of components for their products that are sold in the consumer market (Fig 1a), we hypothesize that vertically integrated firm(s) have expanded their component production beyond only supplying for their own end-products and have taken up key suppliers roles, thus leading to the emergence of coopetition. Specifically, such firms are able to engage in cooperative component supplier relationship with other end-product manufacturers while competing with their products in the consumer market (Fig 1b). This is the case in the aforementioned Apple—Samsung example, where the vertically integrated firm, Samsung, supplied components to its competitor, Apple, resulting in coopetitive behavior. Interestingly, the strategic intent, which usually describes typical coopetitive relationships, of such behavior is not obvious.

There are only a handful of quantitative works [14–16] that describe such behavior, mostly by applying the game theoretical approach. In particular, for supply chain coopetition, Gurnani et al. [17] investigated different decision making structures of the supplier’s product quality investment and buyer’s selling effort, and found the optimal configuration for each party. A two-stage game of coopetition was also developed to study the supply chain alliance formation amongst competitors [18]. However, the supplier-buyer type of relationship considered in the former study did not capture the competitive aspect of coopetition, while the latter had limited its scope up to 3 firms. In addition, both of works did not consider the entire supply chain network dynamics and lacked practical methods to quantify and elucidate coopetitive relationships. Unlike game theory models, supply chain coopetition was introduced within the optimization model, minimizing total supply chain cost through buyer cooperation and suppliers competition [19]. However, this single buyer—multiple suppliers’ model failed to represent the actual behavior of coopetition whereby entities interact with each other competitively and cooperatively at the same time.

In this study, we developed a mathematical model to describe manufacturing supply chain coopetitive relationship through a set of novel constraints that uses a quantification term called coopetition factor. To the best of our knowledge, this is the first model to consider the role of vertically integrated firms on the emergence of supply chain coopetition. It allows us to simulate the product manufacturer-component supplier relationship of multiple agents in the supply chain network and produce optimal coopetitive strategies while satisfying predicted consumer demand. The utility and practicality of the model are demonstrated using a case study based on a hypothetical supply chain network on smartphone manufacturing under different scenarios, thus proposing their strategically viable optimal interactions.

## Model Formulation

The manufacturing supply chain relationship can be mathematically formulated as a MINLP model to determine component procurement contracts of time period *T* for each end-product manufacturing company from various component suppliers in a supply chain network. Set *I* contains all the different end-product manufacturing companies in the particular industry, set *J* has the different types of components involved in the end-product, set *K* is the group of component suppliers involved in the supply chain network and set *L* consists of the model variants for each type of components.

The notations used in the model are as follows:

### Indices

- *i*, end-product manufacturing company
- *j*, type of component
- *k*, component supplier
- *l*, component model variant

### Parameters

- *w_coop_*, weight scaling the importance of the coopetition term relative to the cost
- *w_comp_*, weight scaling the importance of the competition term relative to the cost
- *minOrder_jk_*, minimum order quantity of component type *j* from supplier *k*
- *prodLimit_jk_*, supplier *k*’s production limit of component type *j*
- *Demand_ijl_*, manufacturer *i*’s demand for component variant *l* of type *j*
- *m_jkl_*, gradient of supplier *k*’s pricing function for component variant *l* of type *j*
- *c_jkl_*, y-intercept of supplier *k*’s pricing function for component variant *l* of type *j*
- *e_ijl_*, marketing effort made by the manufacturer *i* for end-product with variants *l* for components type *j*
- *θ_ijl_*, perceived quality (e.g. build quality, software interface) of manufacturer *i*' s end-product with variants *l* for components type *j*
- *α*, value of the market
- *p_ijl_*, price of manufacturer *i*' s end-product with variants *l* for components type *j*
- *γ_i_*, effectiveness of the manufacturer *i*’s marketing on demand;
- *λ_i_*, effect of perceived quality on demand
- *ξ*, noise term of mean 0 and standard deviation σ
- *q_jkl_*, quality of component variant *l* of type *j* from supplier *k*
- *Quality_ijl_*, manufacturer *i*’s minimum quality requirement for component variant *l* of type *j*

### Binary Variables

- *x_ijk_*, 1 when end-product manufacturer *i* purchases component *j* from supplier *k*; 0 otherwise

### Continuous Variables

- *TotalCost*, sum of the industry’s end-product manufacturers’ cost
- *CoopCost*, sum of *TotalCost* with a weighted coopetition term
- *CompCost*, sum of *TotalCost* with a weighted competition term
- *coop_i_*, coopetition factor
- *numComp_ijkl_*, number of component variant *l* of type *j* that company *i* purchases from supplier *k*
- *Price_ijkl_*, price of component variant *l* of type *j* that company *i* pays to supplier *k*

### Economic objective function for supply chain simulation

The supply chain is the cost driver of the end-product manufacturer and does not affect its revenue. Hence, every firm wants to minimize its costs. However, there is limited availability of components available for contract within the supply chain network because suppliers do not have excessive capacity beyond their typical demand. Thus, the firms’ ideal lowest cost, which occurs when there are no bidders for the supplies in the network, may not be attainable due to the cost minimization of their competitors as illustrated later in section “Measuring the coopetition factors”. For example, firm A purchases all the components from supplier C, thus firm B is not able to purchase from supplier C and have to buy from other suppliers. Therefore, in order to simulate the contractual relationships in the supply chain network, the sum of the end-product manufacturers’ cost is minimized. In addition, firms, which pay suppliers a higher monetary value, will have higher buyer’s influence over its suppliers to reduce its overall cost and availability of components (e.g. Apple). This is reflected in the objective function, where the firm with the larger cost will undergo more minimization compared to another company with a smaller cost.

$$\text{min}TotalCost=\sum_{i\in I}\sum_{j\in J}\sum_{k\in K}\sum_{l\in L}\left(numComp_{ijkl}*Price_{ijkl}\right)$$ (1)

### Coopetition factor

**Fig 2:**
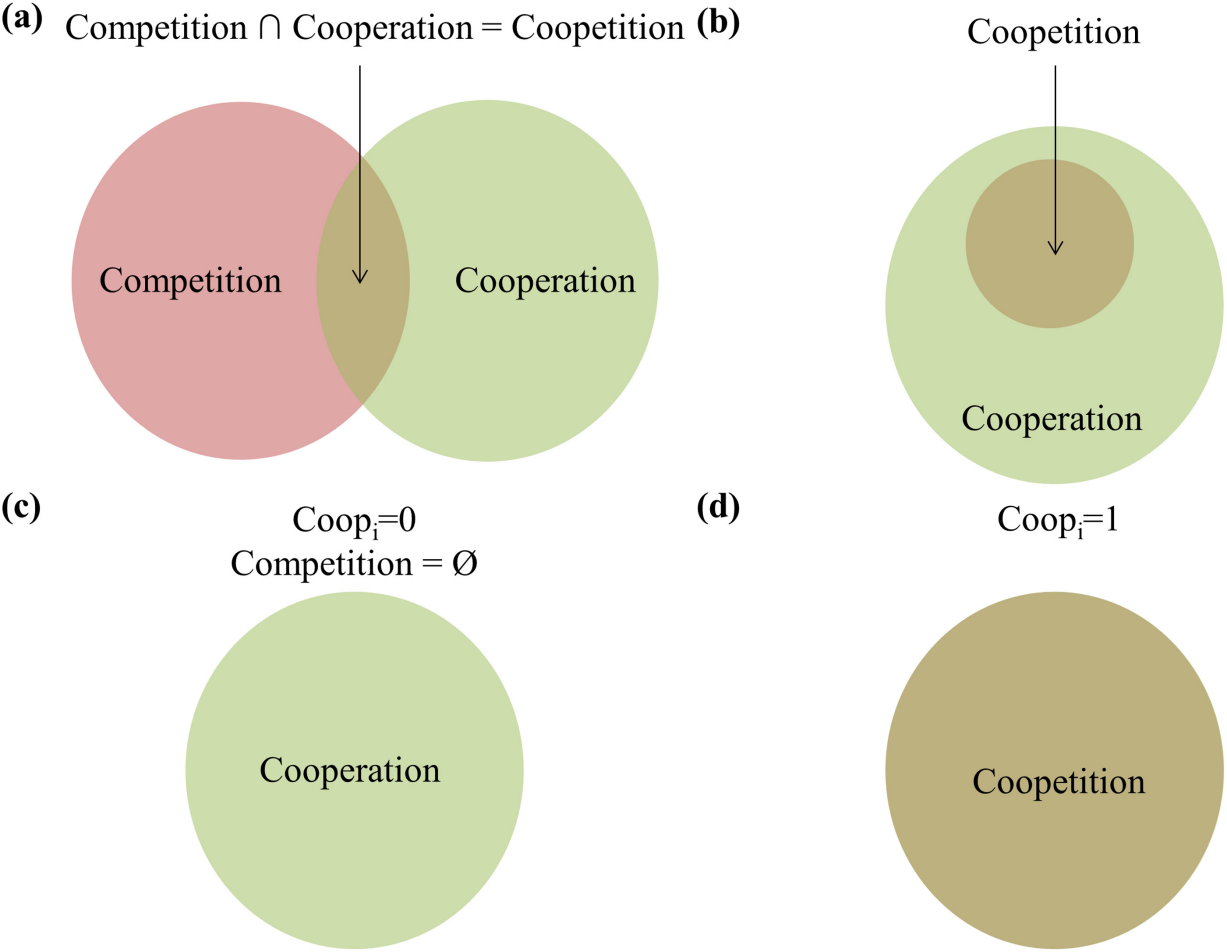
Classifying manufacturers’ interactions with their suppliers. (a) In general, there are competitive and cooperative interactions and their intersection forms coopetitive interactions; (b) In the manufacturing supply chain, the competitive interactions only occur together with cooperation interactions; (c) When *coop*_*i*_ = 0, there are only cooperative interactions; (d) When *coop*_*i*_ = 1, interactions are simutaneously competitive and cooperative, leading to purely coopetitive interactions.

In order to quantify the degree of coopetition, where cooperation and competition amongst different entities coexist, we introduce a coopetition factor (*coop*_*i*_) to measure the willingness of company *i* to cooperate with its competitors. Conceptually, it is defined based on a quantifiable term, which in the case of supply chain relationships is value (V) of goods traded, of the competitive and cooperative interactions (Fig 2a) of company *i* with other firms:

$$coopetitionfactorfori=\frac{V(competition_{i}\cap cooperation_{i})}{V(competition_{i}\cup cooperation_{i})}=\frac{V(coopetition_{i})}{V(allinteractions_{i})}$$ (2)

Applying this concept to the end-product manufacturers in the supply chain, the coopetition factor is defined as the fraction of total cost that is involved in the coopetitive relationship. For example, if 40% of the total cost of components for company *i* is purchased from fellow coopetitors, *coop*_*i*_ equals to 0.4. However, if company *i* is a vertically integrated company, the arrangement of supplying its components for its own end-products will not be considered as coopetition because it does not constitute to inter-firm relationship.

$$coop_{i}=\frac{\sum_{j\in J}\sum_{k\in \{K\cap I\},\;k\neq i}\sum_{l\in L}(numComp_{ijkl}*Price_{ijkl})}{\sum_{j\in J}\sum_{k\in K}\sum_{l\in L}(numComp_{ijkl}*Price_{ijkl})}\;\forall i\in I$$ (3)

where *K∩I* consists of firms that are both component suppliers and end-product manufacturers, i.e. potential coopetitors; *k ≠ i* excludes supplier *i* of vertically integrated firm *i*.

Pure competition is not possible between buyer and supplier (Fig 2b) due to the cooperative nature of their relationship. Hence, when the coopetition factor equals to zero, firm *i* does not cooperate with its competitors and only forms cooperative relationships with non-vertically integrated suppliers (Fig 2c). On the other hand, when coopetition factor equals to one (Fig 2d), maximum coopetition for manufacturer *i* is achieved by purchasing all its components from its competitors. The coopetition factor, for each end-product manufacturing company, will be applied as a set of constraints while the other constraints model the manufacturing supply chain relationship.

### Supply Chain Constraints

#### Binary variable *x*

The binary variable x_ijk_ is defined as follows:

$$\begin{array}{l} x_{ijk}={\{}_{\text{ 0, otherwise}}^{\text{ 1, if end-product manufacturer }i\text{ purchases component }j\text{ from supplier }k}\;\\ \forall i\in I,j\in J,k\in K \end{array}$$ (4)

In some cases, companies are vertically integrated and manufacture some of their own components, which will be used in their own products as well as for sale to other companies. Hence, when supplier *k* and manufacturer *i* are from the same firm (*k = i*),

$$x_{iji}={\{}_{\text{ 0, otherwise}}^{\text{ 1, if supplier }i\text{ produces component }j}\;\forall i\in \{I\cap K\},j\in J$$ (5)

#### Order quantity constraint

The supplier imposes a minimum quota on the manufacturer’s order to ensure a basic level of economies of scale. On the other hand, the manufacturer cannot purchase beyond the supplier’s production limit. The number of components from each supplier is determined as follows:

$$minOrder_{jk}*x_{ijk}\leq \sum_{l\in L}numComp_{ijkl}\leq prodLimit_{jk}*x_{ijk}\;\forall i\in I,j\in J,k\in K$$ (6)

#### Production limit

The maximum number of components each supplier is able to sell to all its buyers is at its production capacity limit. The supplier is assumed to have flexible manufacturing capabilities [20] whereby they can easily modify component production parameters to manufacture a range of component variant *l* of type *j* to meet end-manufacturer requirements.

$$\sum_{i\in I}\sum_{l\in L}numComp_{ijkl}\leq prodLimit_{jk}\;\forall j\in J,k\in K$$ (7)

#### Manufacturing requirement

The company *i* must be able to purchase enough components in order to meet their end-product manufacturing demands for each component variant *l* of type *j*.

$$\underset{k\in K}{\sum }numComp_{ijkl}\geq Demand_{ijl}\;\forall i\in I,j\in J,l\in L$$ (8)

The demand for their end-products faced by manufacturer *i* can be determined from the function formulated by [17] or other similar demand functions:

$$Demand_{ijl}=\alpha -p_{ijl}+\gamma_{i}e_{ijl}+\lambda_{i}\theta_{ijl}+\xi$$ (9)

#### Component pricing

Through the economies of scale, the pricing of each component variant *l* of type *j* that supplier *k* charges company *i* decrease with increasing the quantity ordered. This is taken to be a linear function.

$$Price_{ijkl}=m_{jkl}*numComp_{ijkl}+c_{jkl}\;\forall j\in J,k\in K$$ (10)

#### Quality requirement

End-product manufacturer *i* requires the components from supplier *k* to meet a certain level of requirement.

$$q_{jkl}\geq Quality_{ijl}*x_{ijk}\;\forall i\in I,j\in J,k\in K,l\in L$$ (11)

## Results and Discussion

**Fig 3:**
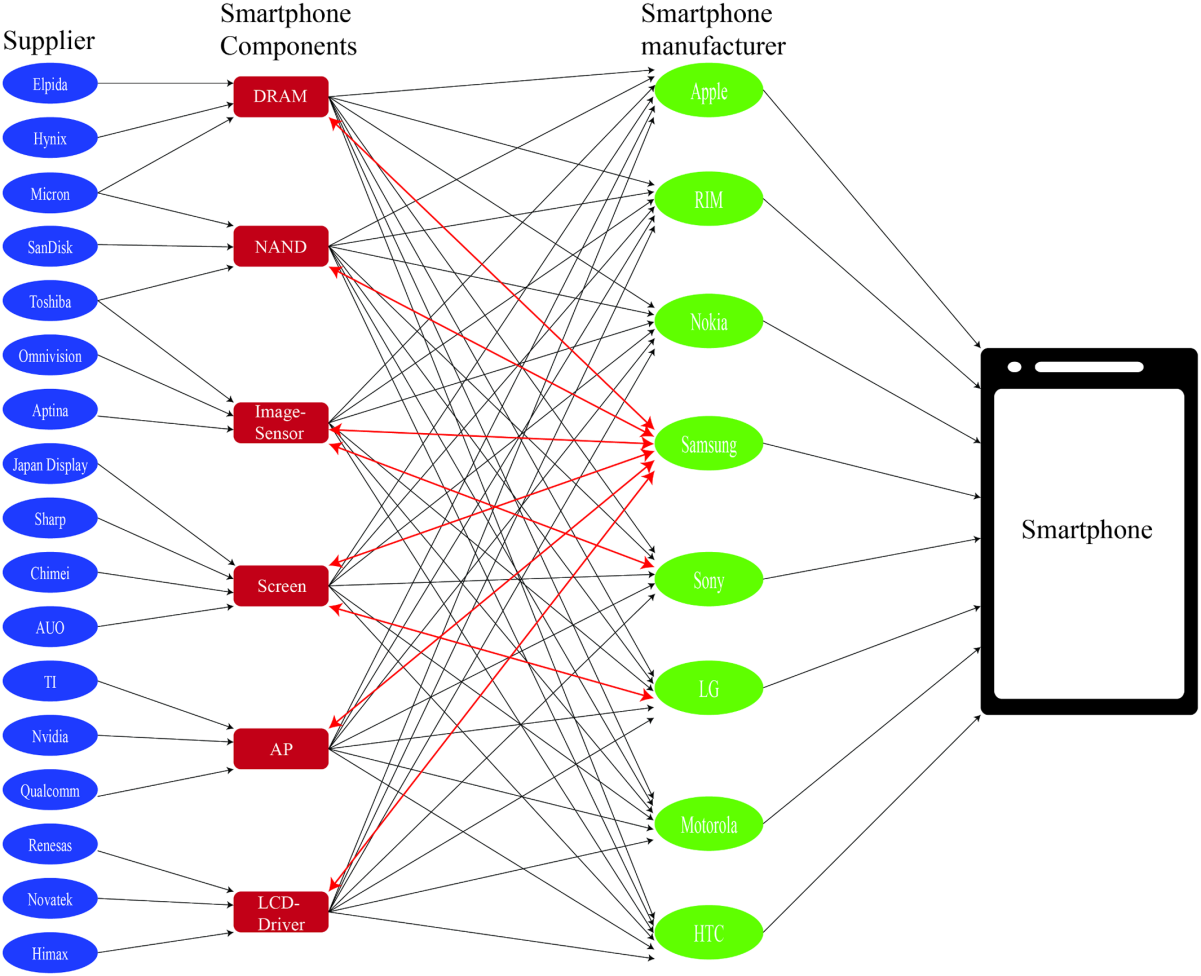
Smartphone supply chain network. Some of the smartphone manufacturing firms are also component suppliers such as Samsung, LG and Sony; Red bidirectional arrows point to the components which vertically integrated firms produce and sell to other manufacturers.

As a case study, the current supply chain model was applied to a hypothetical smartphone manufacturing network (Fig 3) since Samsung, LG and Sony are vertically integrated companies and Samsung is the largest component supplier for NAND flash, display, DRAM and LCD driver IC. Thus, this case study is suitable to validate the hypothesis mentioned in the introduction. The model was formulated as a mixed integer non-linear programming (MINLP) problem in the GAMS environment and solved using Baron [21]. The model parameter values can be found in Tables 1–4. The smartphone manufacturing supply chain mainly includes smartphone end-product companies, *I* (Apple, HTC, LG, Motorola, Nokia, RIM, Samsung and Sony), smartphone components, *J* (NAND, Screen, AP, DRAM, Image-sensor, LCD-Driver), and component suppliers, *K* (Samsung, Toshiba, SanDisk, Micron, JapanDisplay, LG, Sharp, Chimei, AUO, Qualcomm, TI, Nvidia, Hynix, Elpida, Omnivision, Aptina, Sony, Renesas, Novatek, Himax and others). As the parameters for demand was available, Eq (9) was not used. Due to the limited publically available information, set *L* for model variants of different component types and Eq (11) for quality requirements cannot be considered. In addition, as smartphone manufacturing supply chains are highly globalized, we can assume that quality of components from different suppliers are similar. This is because suppliers face global competition from other suppliers of the same type component. Hence, they will try their best to match each other's price and quality. As there is no coopetition in the assembly process, it is not considered in the supply chain network. The contract period *T* in this case study is 1 year.

**Table 1:** Model parameters for indices and *minOrder*_*j*,*k*_ [22].

Parameter	Value
*i*	Apple, HTC, LG, Motorola, Nokia, RIM, Samsung, Sony
*j*	NAND, Screen, AP, DRAM, Image-sensor, LCD-Driver
*k*	Samsung, Toshiba, SanDisk, Micron, JapanDisplay, LG, Sharp, Chimei, AUO, Qualcomm, TI, Nvidia, Hynix, Elpida, Omnivision, Aptina, Sony, Renesas, Novatek, Himax, Others
*minOrder*_*j*,*k*_	= 1 if prodLimit_j,k_ is positive;
	= 0 if prodLimit_j,k_ is 0.

**Table 2:** Model parameter values of *m*_*jk*_ and *c*_*jk*_ [22].

Component types (*j*)	For all *k*	
	*m*_*jk*_	*c*_*jk*_
NAND	-0.0227	22.0227
Screen	-0.0227	20.0227
AP	-0.0227	17.0227
DRAM	-0.0227	10.0227
Image-sensor	-0.0114	2.0114
LCD-Driver	-0.0114	2.0114

**Table 3:** Model parameter values of *Demand*_*i*_ (in mn) [22].

Manufacturer (*i*)	*Demand*_*i*_
Apple	89
Samsung	87
Nokia	85
RIM	52
HTC	43
LG	19
Motorola	17
Sony	20

**Table 4:** Model parameter values of *prodLimit*_*j*,*k*_ (in mn) [22].

Suppliers (*k*)	*prodLimit*_*j*,*k*_ for component types (*j*)					
	NAND	Screen	AP	DRAM	Image-sensor	LCD-Driver
Samsung	146.32	118	56.64	179.36	118	141.6
Toshiba	127.44				37.76	
SanDisk	89.68					
Micron	42.48			61.36		
JapanDisplay		94.4				
LG		70.8				
Sharp		47.2				
Chimei		47.2				
AUO		47.2				
Qualcomm			165.2			
TI			94.4			
Nvidia			23.6			
Hynix				99.12		
Elpida				75.52		
Omnivision					141.6	
Aptina					94.4	
Sony					56.64	
Renesas						94.4
Novatek						70.8
Himax						47.2
Others	6.08		72.16			58

### Measuring the coopetition factors

In order to measure the coopetition factors of various smartphone manufacturers, their existing supply chain relationship and order allocations are required (see Eq 3). Hence, using various bills of materials (BOMs) of different products from each smartphone manufacturer (S1 Table), the related supply chain relationship binary variables *x*_*ijk*_ were set to 1. The iPhone’s components suppliers were limited to those reported in the BOM as teardowns of the iPhone and its BOM were actively conducted and documented. However, this was not the case for the other smartphone manufacturers, which rarely had teardowns and reports of their BOMs, hence there may be a possibility of other suppliers not documented in the BOM. Therefore, in addition to the suppliers in their BOMs, they were allowed to form supply chain relationships with other suppliers. Using these information, the model estimated the component allocations as well as each smartphone manufacturers’ coopetition factor.

Furthermore, to study the changes of coopetition factor with respect to time, simulations were conducted for two different time periods: T1 and T2, where iPhone 4s and iPhone 5 were Apple’s flagship smartphone at that time respectively. As there was a change of suppliers from the iPhone 4s to iPhone 5, Apple’s supply chain relationships were changed accordingly for each time period in the model while the suppliers in the BOMs of other smartphone manufacturers remain unchanged.

**Fig 4:**
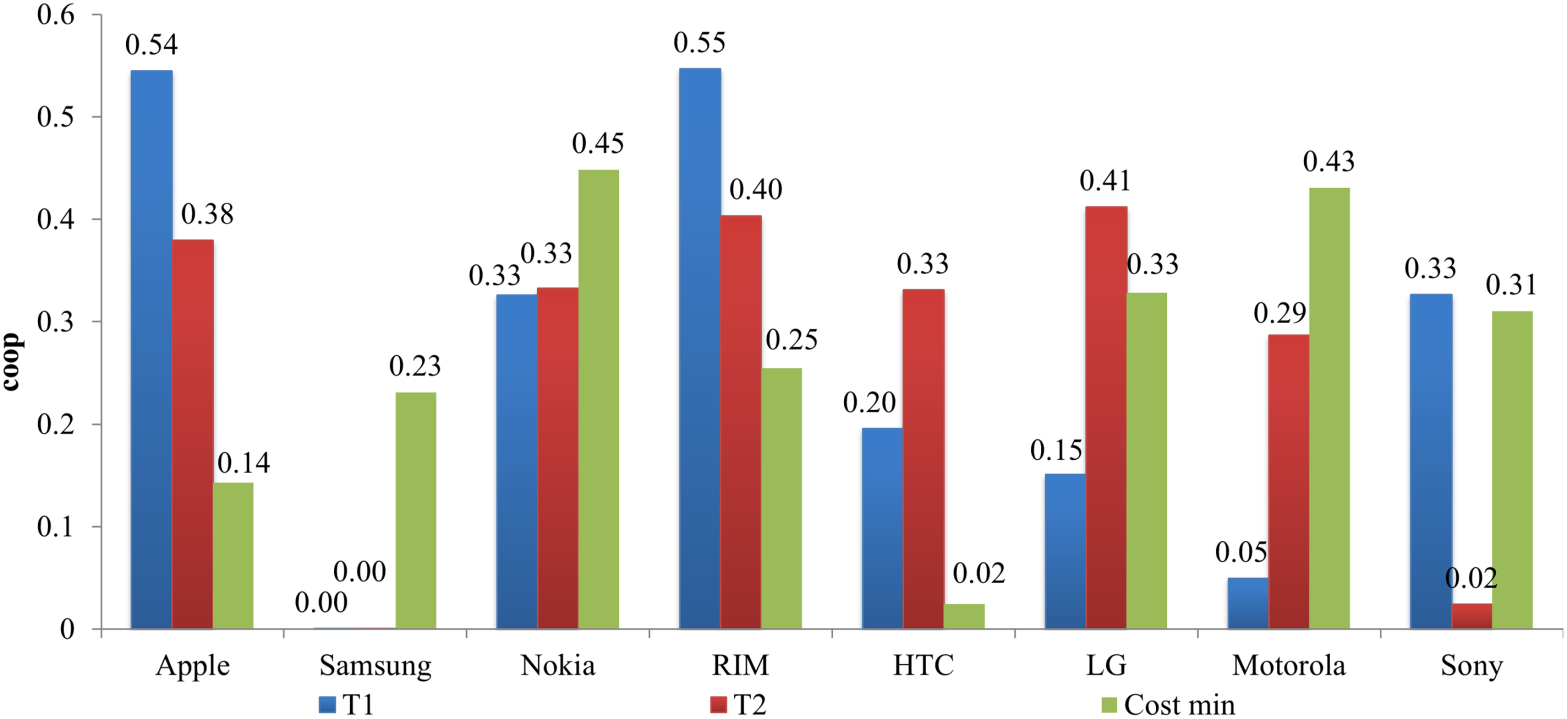
Coopetition factors (*coop*_*i*_) for each smartphone manufacturer.

The coopetition measure for both time periods were low (Fig 4) and this can be observed by the fierce competition and lack of collaborative efforts with competitors in the industry. This is could be due to the limited advantages of coopetition with vertically integrated companies compared to cooperating with other suppliers. The coopetition factor for Apple significant drops from 0.545 in T1 to 0.380 in T2 (Fig 4) because of the removal of Samsung from the list of display suppliers and addition of SanDisk, which is not a co-opetitor, to the NAND suppliers (S1 Table). These were the two most expensive components in smartphones which resulted in a major decrease in total monetary value of Samsung components purchased by Apple. This possibly reflected the worsening relations between the two firms, e.g. lawsuit on infringement of patents [23]. On the other hand, Samsung’s coopetition factor was near zero because Samsung manufactured most of its components, as a result it was mostly self-sufficient.

Despite maintaining the same suppliers in the BOMs for the other firms, their coopetition factors changed for two different time periods (Fig 4): the decrease on Apple’s purchase of Samsung components leads to the respective increase and decrease in available Samsung and other supplier components to other companies, resulting in changes to coopetition factors in this model. Thus, this clearly indicated the impact of one firm’s actions on the others as stated earlier in the “Economic objective function” as well as the non-static nature of coopetition because it varies with changes in supply chain interactions.

### Best case minimum cost, coopetition friendly and adverse scenario simulation

To determine the minimum overall cost of manufacturers (which is the best case scenario), the model was applied to the smartphone supply chain network without considering existing relationships and the coopetition factors obtained earlier. Nokia and Motorola had the largest coopetition factors of 0.448 and 0.431 respectively while HTC had the smallest coopetition factors of 0.025 (Fig 4). The best case minimum (Table 5), which was lower compared to T1 and T2, was achieved through the decrease in cost for Apple, RIM, HTC and Motorola. However, other firms would incur increased cost which would conflict with their own interests, thus posing some difficulty to shift from T2 to the best case scenario. It might also be possible that some manufacturers could only make satisficing decisions due to their limited information, cognitive capabilities and time, which is known as bounded rationality [24, 25], hence giving rise to a sub-optimal scenario.

**Table 5:** Cost for each smartphone manufacturer in various scenarios.

Scenario	Cost for smartphone company ($)								Total Cost
	Apple	Samsung	Nokia	RIM	HTC	LG	Motorola	Sony	
T1	5687	5514	5566	3519	2942	1352	1221	1424	27225
T2	5740	5546	5452	3539	2963	1353	1217	1419	27227
Cost min	5606	5567	5463	3525	2963	1353	1213	1422	27111

To simulate the coopetition friendly and coopetition adverse market scenarios, the sum of the coopetition factors were maximized and minimized respectively while simulating the supply chain economic objective, thus resulting in bi-objective optimization. In the coopetition friendly setting, the companies favor cooperating with their competitors. Thus, coopetition was maximized through minimizing the negative sum of the coopetition factors.

$$\text{min}Coop\text{Cos}t=\sum_{i\in I}\sum_{j\in J}\sum_{k\in K}(numComp_{ijk}*Price_{ijk})-{\text{w}}_{\text{coop}}\sum_{i\in I}coop_{i}$$ (12)

On the other hand, in a coopetition adverse setting, firms are less willing to cooperate with their competitors but instead favor other suppliers that were not involved in the upstream market. Hence, coopetition was minimized through the positive sum of the coopetition factors.

$$\text{min}Comp\text{Cos}t=\sum_{i\in I}\sum_{j\in J}\sum_{k\in K}\left(numComp_{ijk}*Price_{ijk}\right)+{\text{w}}_{\text{comp}}\sum_{i\in I}coop_{i}$$ (13)

**Fig 5:**
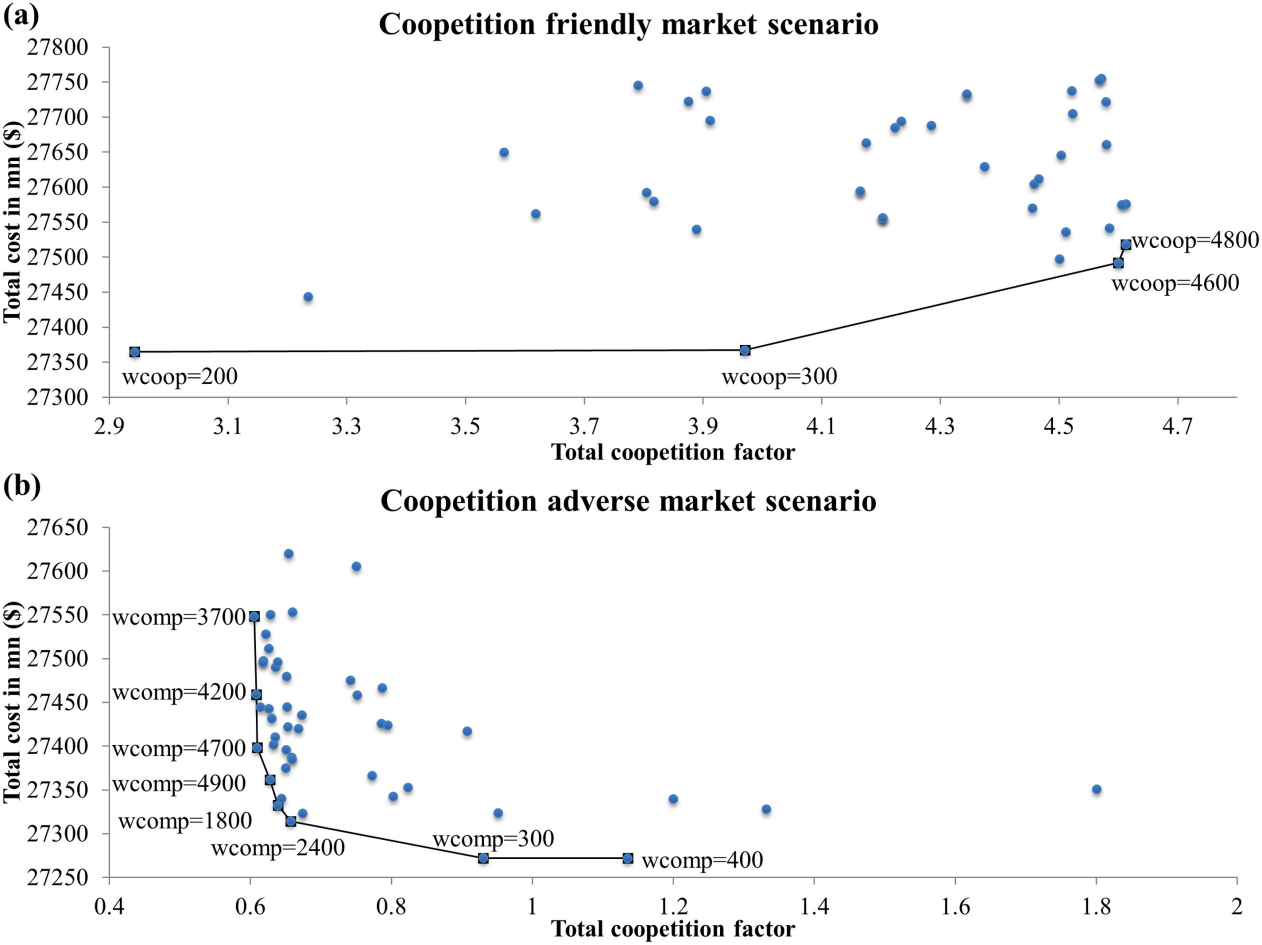
Bi-objective optimization Pareto front. (a) Coopetition friendly; and (b) coopetition adverse market scenario.

For bi-objective functions Eqs (12) and (13), the weights were varied from 100 to 5000 and the Pareto front for each market scenario were plotted (Fig 5). In the coopetition friendly market scenario, the total end-product cost increases with increasing total coopetition factor. On the other hand, the total cost increases with decreasing total coopetition factor in the coopetition adverse market scenario. Hence, these scenarios are unlikely to occur and a certain level of coopetition is favorable. The total cost increases for both market situations compared to T1 and T2. Thus, the current market is most likely coopetition neutral. The smallest total coopetition factor in the Pareto front of the competitive scenario is 0.605. Therefore, coopetition is evitable due to key supplier roles that vertically integrated companies, Samsung, LG and Sony, have taken up.

### Optimal coopetitive supply chain for Apple, Samsung, Nokia and Motorola

Based on the model, the end-product manufacturer is able to determine the optimal level of coopetition to engage in and its respective supply chain configuration. The ideal scenario for a manufacturer is to solely minimize its own cost as the objective function. However, as mentioned in the “Economic objective function”, this scenario occurs when there are no bidders for the supplies in the network, which may not be attainable due to the cost minimization of competitors. Despite being unrealistic, the ideal scenario is the most desired outcome for the manufacturer, thus it will be used as a benchmark.

On the other hand, a realistic strategy for the end-product manufacturer will be to minimize the overall cost of the industry while varying the coopetition factor of firm in consideration. The one that yields the lowest cost for that company is the optimal level of coopetition. For both methods, an additional constraint is added such that Apple will not purchase its components from Samsung because their relationship was reported to be worsening.

In general, both strategies produced costs that are lower than T1 and T2 (Table 5) as such they are favorable for the respective firms. The maximum feasible coopetition factor for the realistic strategy increases (Table 6) with decreasing component quantity requirements because it was easier for coopetitiors to fulfill. There are 3 coopetitive suppliers—LG, Sony and Samsung, where Samsung is involved with most of the component types. Therefore, coopetition is most likely to benefit the component supplier division of Samsung. The supply chain configurations and respective coopetition factors of the realistic strategy will be compared against the ideal scenario to its viability.

**Table 6:** Range of feasible *coop*_*i*_ values for realistic strategies.

Company (*i*)	Range of feasible *coop*_*i*_ values	
	Min	Max
Apple	0	0.238
Samsung	0	0.241
Nokia	0	0.917
Motorola	0	1

**Fig 6:**
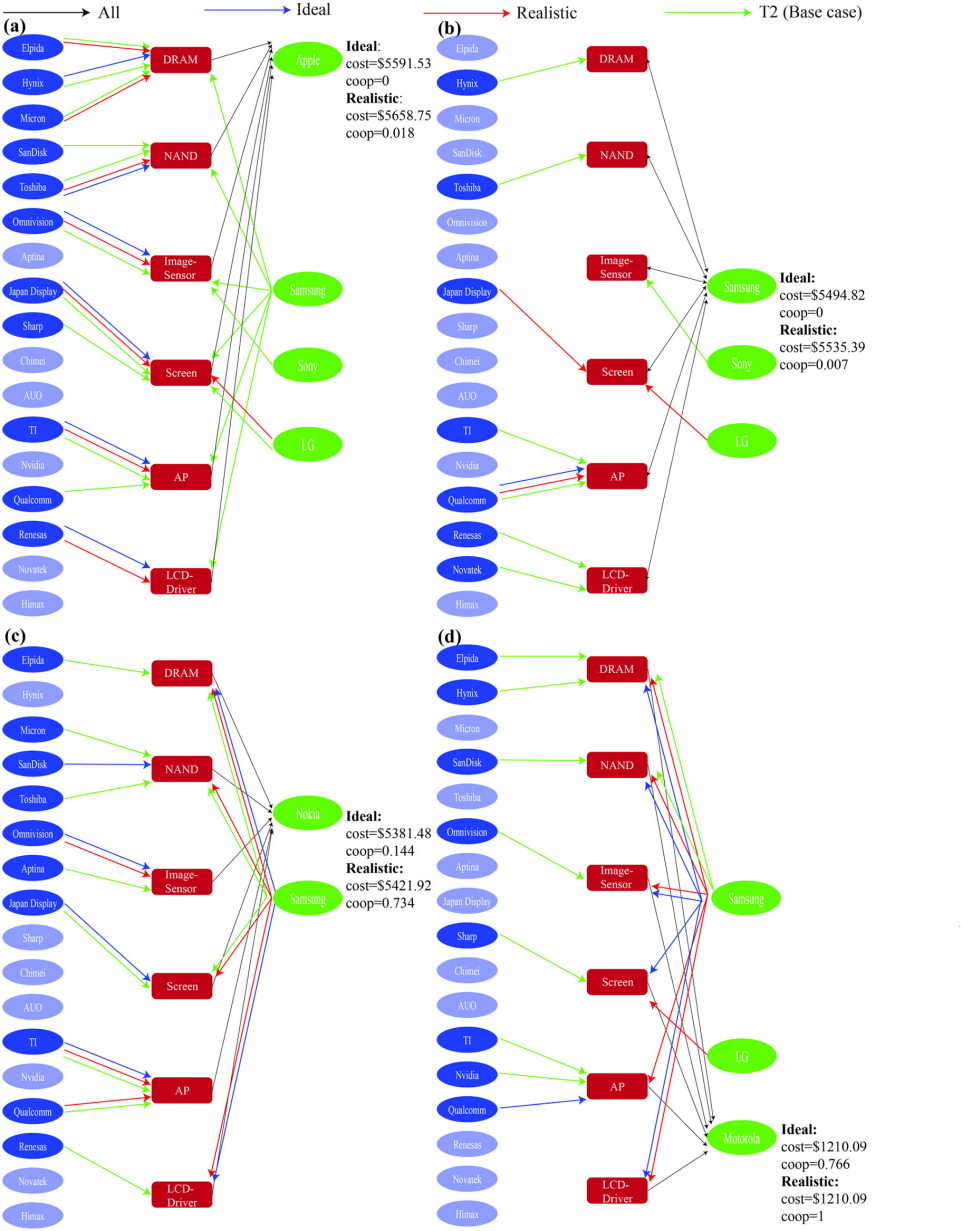
Supply chain network for ideal, realistic and theoretical strategies. (a) Apple; (b) Samsung; (c) Nokia; and (d) Motorola.

Comparing the different supply chain configurations for Apple, the differences between the ideal scenario and realistic strategy were the addition of LG as screen supplier and replacement of Hynix's DRAM supplier role by Micron and Elpida (Fig 6a). Moreover, the coopetition factors of both methods were relatively close with zero and 0.018 for the ideal scenario and realistic strategy respectively, thus it is possible for Apple to reduce its reliance on its coopetitors’ especially Samsung. Due to its similarity to the ideal scenario, the realistic strategy could be a highly possible plan for Apple to adopt.

In Samsung’s case, the optimal coopetition factor for the ideal scenario and realistic strategy were 0 and 0.007 respectively (Fig 6b) because Samsung produced its own components, which made purchasing components from other competitors more costly. In the ideal scenario, most of the components were supplied internally within Samsung other than the application processor, which was supplied by Qualcomm. This had similar resemblance to the year 2013 market situation, where Samsung’s Exynos and Qualcomm’s Snapdragon processor chip were used in two variants of its Galaxy S4 smartphone [26]. In terms of supply chain configuration, the realistic strategy added LG and Japan Display as screen suppliers. Given the close similarities between both methods, the realistic strategy might be a good prospect for Samsung to follow.

Nokia had a wide range of viable coopetition factors from 0 to 0.917 (Table 6) for the realistic strategy because its coopetitors were able to fulfill most of Nokia’s relatively low component requirements. The optimal coopetition factors for ideal and realistic methods differed significantly (Fig 6c) while the increase in cost followed the similar trend to Apple (Fig 6a) and Samsung (Fig 6b). In terms of supply chain configuration, Samsung had a more coopetitive role in the realistic strategy, where it replaced SanDisk and Japan Display to supply the NAND and screen respectively. However, their similar cost indicates Nokia’s possible indifference to coopetition as such suppliers can be easily substituted with non-coopetitive ones and viability of the realistic strategy.

Due to its relatively smaller component requirements, Motorola was able to vary the coopetition factor through the whole range for the realistic strategy (Table 6). The ideal and realistic methods had high coopetition factors of 0.766 and 1 respectively while having the same costs. The main coopetitor for Motorola was Samsung, which supplied most of the types of components for both cases (Fig 6d). The only difference was that Qualcomm supplied the application processor in the ideal scenario while LG supplied the screen for the realistic strategy. Therefore, the realistic strategy could be easily implemented and achieve the same costs as the ideal scenario unlike the other three firms.

## Conclusion

In this paper, an MINLP supply chain coopetition relationship model is developed. The coopetition factor is introduced in the supply chain network model to quantify the level of coopetition the firm is involved. This model is applied to a case study of a hypothetical smartphone industry. The coopetition factors of each manufacturing firm were measured and showed that coopetition levels are moderately low in the smartphone industry. Different market scenarios have shown that coopetition is inevitable and a right mix of coopetition is optimal for the industry. The realistic method exhibits similar properties with the ideal approach and is viable choice. Clearly, this model can be further exploited for consideration in market policy regulations and also herald a new era of global coopetitive business.

## Supporting Information

S1 Table Component suppliers for each end-product smartphone company. (DOC)
