# Supplementary material for: Coopetitive Supply Chain Relationship Model: Application to the Smartphone Manufacturing Network
Source: PLoS One. 2015 Jul 17;10(7):e0132844. doi: 10.1371/journal.pone.0132844 (PMC4506052; doi:10.1371/journal.pone.0132844)
Supplement: S1 Table — (DOC) [file pone.0132844.s001.doc]

**Table S1.** Component suppliers for each end-product smartphone company.

| Smartphone companies (*i*) | Supplier for Component Types (*j*) | | | | | Ref |
| --- | --- | --- | --- | --- | --- | --- |
|  | NAND | Display | AP | DRAM | Image sensors |  |
| HTC | Samsung | - | Qualcomm  Nvidia | Samsung | - | [1, 2] |
| LG | Samsung | - | TI  Qualcomm  Nvidia | - | Aptina | [2-5] |
| Motorola | SanDisk | - | Qualcomm  TI  Nvidia | Samsung  Hynix  Elpida | Omnivision | [1, 2, 6, 7] |
| Nokia | Micron  Toshiba | Samsung | TI  Qualcomm | Samsung  Elpida | Toshiba | [8-10] |
| RIM | Samsung | Samsung | Qualcomm | - | Aptina | [11-14] |
| Samsung | - | - | Qualcomm | - | Sony | [2, 15] |
| Sony | Micron | - | Qualcomm | Elpida | - | [2, 16] |
| Apple’s  iPhone 4s | Samsung  Toshiba | Samsung  LG  Chimei | Samsung | Elpida  Samsung  Hynix | Sony  Omnivision | [17-19] |
| Apple’s  iPhone 5 | Toshiba  Samsung  SanDisk | LG  Sharp  Japan Display | Samsung | Hynix  Elpida  Samsung | Sony  Omnivision | [20, 21] |

**Web references for BOM**

1. Nvidia. *Tegra Super Phones*. Available from: <http://www.nvidia.com/object/tegra-superphones.html>.
2. *Commercial Devices - Qualcomm Developer Network*. Available from: [https://developer.qualcomm.com/devices#/data/device/filter?form_build_id=form-fe433bb330d6c8e6e5a752e81086bc95&form_id=qdn_device_db_search_form&sort_by=&items_per_page=15&current_page=0&text_filter_value=Mfg%252C+Name%252C+or+Model+%2523&manufacturer_filter_values%5B%5D=All&operator_filter_values%5B%5D=All&os_filter_values%5B%5D=All&cpu_filter_values%5B%5D=All&gpu_filter_values%5B%5D=All](https://developer.qualcomm.com/devices#/data/device/filter?form_build_id=form-fe433bb330d6c8e6e5a752e81086bc95&form_id=qdn_device_db_search_form&sort_by=&items_per_page=15&current_page=0&text_filter_value=Mfg%252C+Name%252C+or+Model+%2523&manufacturer_filter_values%5B%5D=All&operator_filter_va).
3. *LG Secret (KF750) Mobile Phone - What's Inside_text - Electronic Products*. Available from: <http://www2.electronicproducts.com/LG_Secret_KF750_Mobile_Phone-whatsinside_text-71.aspx>.
4. Hollister, S. *LG Optimus 3D has dual-core 1GHz OMAP 4 CPU, video codecs up the wazoo*. 2011; Available from: <http://www.engadget.com/2011/02/12/lg-optimus-3d-has-dual-core-1ghz-omap4-cpu-video-codecs-up-the/>.
5. *Optimus 4X HD specifications and reviews*. Available from: <http://www.esato.com/phones/LG-Optimus+4X+HD-909>.
6. Chipworks. *Teardown of the Motorola RAZR Smartphone*. 2011; Available from: <http://www.chipworks.com/blog/recentteardowns/2011/11/18/teardown-of-the-motorola-razr-smartphone/>.
7. Chipworks. *Teardown of the Motorola Droid 3*. 2011; Available from: <http://www.chipworks.com/blog/recentteardowns/2011/09/01/teardown-of-the-motorola-droid-3/>.
8. *Nokia Lumia 900 - What's Inside_text - Electronic Products*. Available from: <http://www2.electronicproducts.com/Nokia_Lumia_900-whatsinside_text-128.aspx>.
9. Carav. *Inside the Nokia 808 PureView Smartphone – 41 MP Camera!* 2012; Available from: <http://www.chipworks.com/blog/recentteardowns/2012/07/19/inside-the-nokia-808-pureview-smartphone-with-a-41-mp-camera/>.
10. Rassweiler, A. *Nokia Lumia 900 Carries Bill of Materials of $209* 2012; Available from: <http://www.isuppli.com/Teardowns/News/Pages/Nokia-900-Carries-Bill-of-Materials-of-$209.aspx>.
11. *Blackberry Bold 9700 - What's Inside_text - Electronic Products*. Available from: <http://www2.electronicproducts.com/Blackberry_Bold_9700-whatsinside_text-93.aspx>.
12. *Teardown Report Reveals Manufacturing Cost of BlackBerry Bold*. 2008; Available from: <http://www.cellular-news.com/story/34330.php>.
13. Rassweiler, A. *BlackBerry Torch Carries $171.05 Bill of Materials, iSuppli Teardown Reveals*. 2010; Available from: <http://www.isuppli.com/Teardowns/News/Pages/BlackBerry-Torch-Carries-171-05-Bill-of-Materials-iSuppli-Teardown-Reveals.aspx>.
14. *BlackBerry Storm teardown reveals $203 worth of parts | BGR*. Available from: <http://bgr.com/2009/01/29/blackberry-storm-teardown-reveals-203-worth-of-parts/>.
15. Rwilliamson. *Inside the Samsung Galaxy SIII*. 2012; Available from: <http://www.chipworks.com/blog/recentteardowns/2012/06/01/inside-the-samsung-galaxy-siii/>.
16. TechInsights, U. *Sony Xperia Play Teardown & Analysis*. 2012; Available from: <http://www.ubmtechinsights.com/teardowns/sony-xperia-play-teardown/>.
17. 9To5Mac. *iPhone 4S supply chain explained: The winners and losers*. 2011; Available from: <http://9to5mac.com/2011/10/11/iphone-4s-manufacturing-bom-analysis/>.
18. Chipworks. *iPhone 4S Teardown: A closer look at the chips inside*. 2011; Available from: <http://www.chipworks.com/blog/recentteardowns/2011/10/13/iphone-4s-teardown-a-closer-look-at-the-chips-inside/>.
19. Keller, K. *iPhone 4 Carries Bill of Materials of $187.51, According to iSuppli*. 2010; Available from: <http://www.isuppli.com/Teardowns/News/Pages/iPhone-4-Carries-Bill-of-Materials-of-187-51-According-to-iSuppli.aspx>.
20. Journal, T.W.S. *Why There May Be an iPhone 5 Shortage*. 2012; Available from: <http://blogs.wsj.com/digits/2012/10/08/why-there-may-be-an-iphone-5-shortage/>.
21. Rassweiler, A. *Many iPhone 5 Components Change, But Most Suppliers Remain the Same, Teardown Reveals*. 2012; Available from: <http://www.isuppli.com/Teardowns/News/pages/Many-iPhone-5-Components-Change-But-Most-Suppliers-Remain-the-Same-Teardown-Reveals.aspx>.
